# Genetic Diversity and Reassortment of Hantaan Virus Tripartite RNA Genomes in Nature, the Republic of Korea

**DOI:** 10.1371/journal.pntd.0004650

**Published:** 2016-06-17

**Authors:** Jeong-Ah Kim, Won-keun Kim, Jin Sun No, Seung-Ho Lee, Sook-Young Lee, Ji Hye Kim, Jeong Hoon Kho, Daesang Lee, Dong Hyun Song, Se Hun Gu, Seong Tae Jeong, Man-Seong Park, Heung-Chul Kim, Terry A. Klein, Jin-Won Song

**Affiliations:** 1 Department of Microbiology, College of Medicine, Korea University, Seoul, Republic of Korea; 2 Agency for Defense Development, Yuseong-gu, Daejeon, Republic of Korea; 3 Department of Microbiology, College of Medicine, Institute for Viral Diseases, Korea University, Seoul, Republic of Korea; 4 168th Multifunctional Medical Battalion, 65th Medical Brigade, Unit 15247, 5th Medical Detachment, Yongsan Army Garrison, Seoul, Republic of Korea; 5 65th Medical Brigade, Unit 15281, Public Health Command District-Korea, Yongsan Army Garrison, Seoul, Republic of Korea; CDC, UNITED STATES

## Abstract

**Background:**

Hantaan virus (HTNV), a negative sense tripartite RNA virus of the Family *Bunyaviridae*, is the most prevalent hantavirus in the Republic of Korea (ROK). It is the causative agent of Hemorrhagic Fever with Renal Syndrome (HFRS) in humans and maintained in the striped field mouse, *Apodemus agrarius*, the primary zoonotic host. Clinical HFRS cases have been reported commonly in HFRS-endemic areas of Gyeonggi province. Recently, the death of a member of the ROK military from Gangwon province due to HFRS prompted an investigation of the epidemiology and distribution of hantaviruses in Gangwon and Gyeonggi provinces that border the demilitarized zone separating North and South Korea.

**Methodology and Principal Findings:**

To elucidate the geographic distribution and molecular diversity of HTNV, whole genome sequences of HTNV Large (L), Medium (M), and Small (S) segments were acquired from lung tissues of *A*. *agrarius* captured from 2003–2014. Consistent with the clinical incidence of HFRS established by the Korea Centers for Disease Control & Prevention (KCDC), the prevalence of HTNV in naturally infected mice in Gangwon province was lower than for Gyeonggi province. Whole genomic sequences of 34 HTNV strains were identified and a phylogenetic analysis showed geographic diversity of the virus in the limited areas. Reassortment analysis first suggested an occurrence of genetic exchange of HTNV genomes in nature, ROK.

**Conclusion/Significance:**

This study is the first report to demonstrate the molecular prevalence of HTNV in Gangwon province. Whole genome sequencing of HTNV showed well-supported geographic lineages and the molecular diversity in the northern region of ROK due to a natural reassortment of HTNV genomes. These observations contribute to a better understanding of the genetic diversity and molecular evolution of hantaviruses. Also, the full-length of HTNV tripartite genomes will provide a database for phylogeographic analysis of spatial and temporal outbreaks of hantavirus infection.

## Author Summary

Hemorrhagic Fever with Renal Syndrome (HFRS) and Hantavirus Pulmonary Syndrome (HPS) are endemic zoonotic infectious diseases caused by hantaviruses that belong to the Family *Bunyaviridae* containing negative-sense tripartite RNA genomes. Hantaviruses pose a critical emerging public health threat, with up to 200,000 clinical cases reported annually worldwide with 1–36% case fatality rates. In humans, hantavirus-borne diseases are contracted by the inhalation of viruses aerosolized from rodent excreta. However, there is no effective therapeutic or vaccine to prevent from the disease. Whole genome sequences of Hantaan virus (HTNV) were acquired from lung tissues of *Apodemus agrarius* captured in HFRS-endemic areas of the Republic of Korea (ROK). Phylogenetic analyses demonstrated that sequences of the HTNV tripartite genomes clustered geographically, showing broad diversity of HTNV throughout the areas surveyed. Reassortment analysis first suggested a natural occurrence of the HTNV genetic exchange in the ROK. These observations contribute to a better understanding of the genetic diversity and molecular evolution of hantaviruses in HFRS-endemic regions. The complete sequences of HTNV genomes will provide a database for the phylogeographic analysis and surveillance of endemic hantavirus-borne diseases.

## Introduction

Viruses in the Hantavirus genus of the family *Bunyaviridae* are negative-sense single-stranded RNA virus containing Large (L), Medium (M), and Small (S) segments [1]. Hantaviruses pose an emerging public health threat, with about 200,000 cases of human disease annually worldwide and fatality rates of 1–36% [2, 3]. Old World hantaviruses, e.g., Hantaan virus (HTNV), Puumala virus (PUUV), Seoul virus (SEOV), and Dobrava-Belgrade virus (DOBV), are etiologic agents of Hemorrhagic Fever with Renal Syndrome (HFRS) in Eurasia [4]. In America, Hantavirus Pulmonary Syndrome (HPS) results from infections with New World hantaviruses, e.g., Sin Nombre virus (SNV) and Andes virus (ANDV) [5]. In humans, HFRS and HPS are contracted by inhaling aerosolized infectious particles from rodent salvia, urine, and feces [6]. Hantavirus infections are highly endemic and cause severe diseases in humans. However, there are no effective therapies or vaccines against these viruses.

HTNV is harbored by striped field mice (*Apodemus agrarius*), which constitute about 70% of the wild rodent population in the Republic of Korea (ROK) [7]. HFRS incidences increase during the spring and fall, due to the dynamics of rodent populations [8]. Observance of HFRS cases for military personnel and civilians near the demilitarized zone (DMZ) led to an investigation of the geographic distribution and molecular epidemiology of HTNV in Gangwon and Gyeonggi provinces [9–11]. Over decades, our studies have demonstrated the molecular similarities and diversity of hantaviruses using viral genomic sequences identified from HFRS patients and rodents where the patients were exposed in Gyeonggi province, the highest endemic area in the ROK [8, 9, 12–15]. However, the molecular prevalence of hantaviruses in Gangwon province remains unknown.

Reassortment is a genetic event that results in the exchange of genome segments, and it is a major molecular mechanism to confer genetic diversity [16]. Influenza virus, a negative-sense segmented RNA virus, frequently generates reassortants by switching genomes of viruses from different hosts. Reassortment can play an important role in viral fitness, transmission, and pathogenesis of segmented RNA viruses [17]. The genetic reassortment of hantaviruses has been reported naturally and experimentally [18, 19]. A natural reassortment of SNV within deer mice (*Peromyscus maniculatus*) occurred with the exchange of the M segment. Genetic exchange of the M segment of DOBV was observed between low pathogenic DOBV-Aa and highly pathogenic DOBV-Af *in vitro*. In addition, HTNV strains appeared to be highly divergent in the limited region of Guizhou in China, with the generation of S segment reassortants [20].

In this study, *A*. *agrarius* was collected in Gangwon and Gyeonggi provinces from 2003–2014. To investigate the molecular epidemiology and distribution of HTNV, serological and molecular screening of HTNV was performed from lung tissues of the rodents. Using 34 of complete sequences of HTNV tripartite genomes, phylogenetic analyses show well-supported geographic clusters of the L, M, and S segments in the ROK. Reassortment analysis first demonstrated that HTNV in Dagmar North (DN), Paju, consists of the heterogeneous L segment and homogeneous M and S segments, suggesting that the reassortment of HTNV may occur in nature. This study provides a better understanding of the genetic diversity and molecular evolution of HTNV tripartite genomes in the ROK. The whole genome sequences of HTNV will establish a database for the phylogeographic analysis and surveillance of endemic outbreaks of hantavirus infection.

## Methods

### Ethics statement

Trapping of rodents was approved by US Forces Korea (USFK) in accordance with USFK Regulation 40–1 “Prevention, Surveillance, and Treatment of Hemorrhagic Fever with Renal Syndrome”. Wild rodents were euthanized by cardiac puncture and tissues were dissected under isoflurane anesthesia. All procedures and handling of rodents were conducted under an approved protocol by the Korea University Institutional Animal Care and Use Committee (KUIACUC, #2010–212).

### Rodent trapping

**Fig 1:**
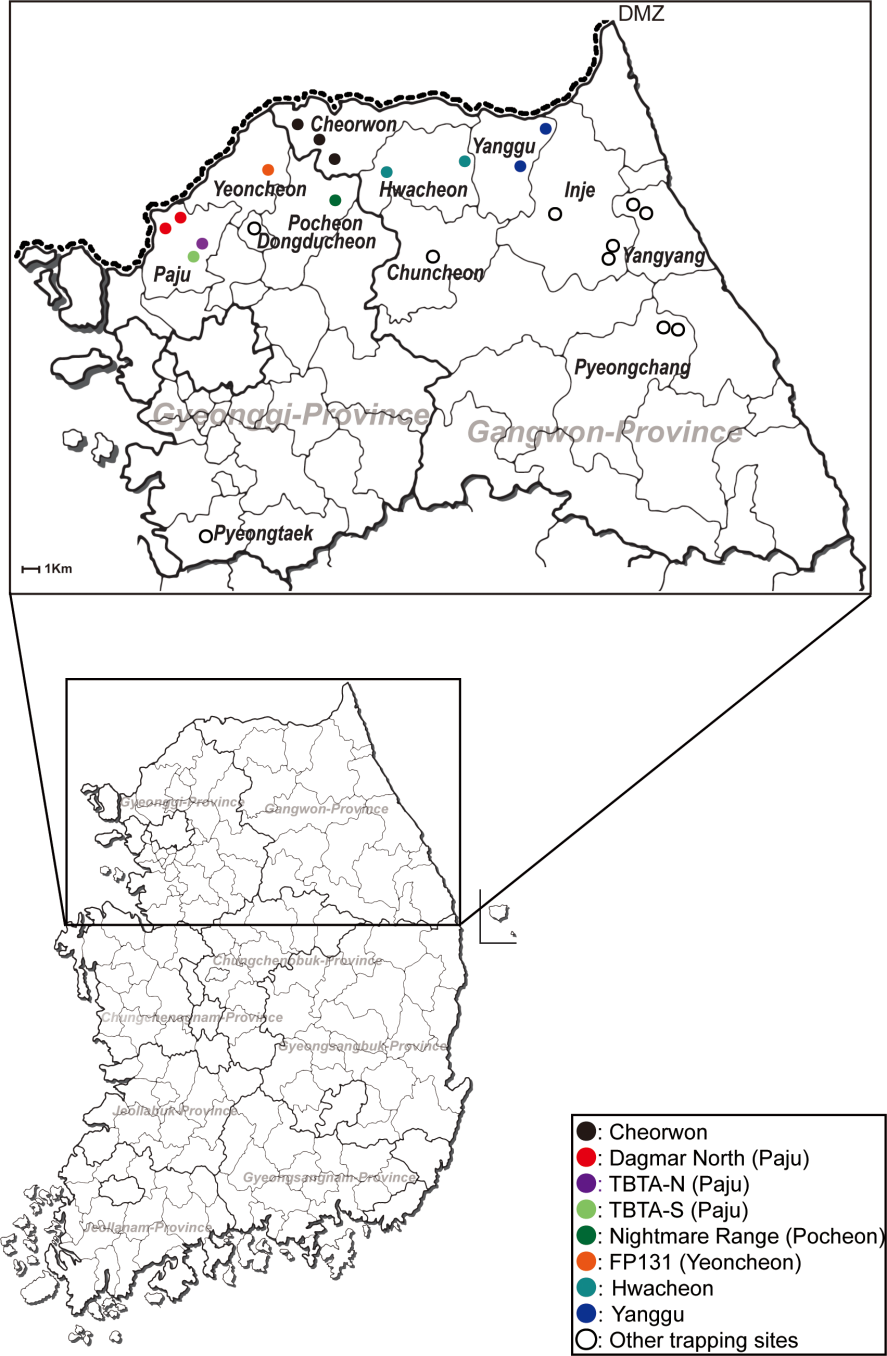
Geographic distribution of trapping sites for *A*. *agrarius* collected in Gangwon and Gyeonggi provinces, the Republic of Korea. A map shows the trapping sites where *A*. *agrarius* was captured from 2003–2014. Color circles indicate the places where the positivity of HTNV was shown by RT-PCR; Dagmar North (DN), red; Twin Bridge Training Area North (TBTA-N), violet; TBTA South (TBTA-S), yellowish green; Nightmare Range (NR), green; Fire Point 131 (FP131), orange; Cheorwon, black; Hwacheon, cyan; opened circles indicate the places where no HTNV RNA was identified.

Rodents were captured in Gangwon and Gyeonggi provinces of the ROK, from 2003–2014 using live-capture Sherman traps (7.7 cm by 9 cm by 23 cm; H. B. Sherman, USA). A total of 100 traps were placed at intervals of about 4 m to 5 m at various US military training areas and ROK civilian sites in Cheorwon, Chuncheon, Hwacheon, Inje, Pyeongchang, Yanggu, and Yangyang in Gangwon province, and Dongducheon, Paju, Pocheon, Phyeongtaek, and Yeoncheon in Gyeonggi province each day over 2–3 consecutive days for each trapping period (Fig 1).

### Immunofluorescence assay (IFA) test

Rodent sera, diluted 1:32, were placed into duplicate acetone-fixed wells of Vero E6 cells infected with HTNV, and the wells incubated at 37°C for 30 min. After incubation, the slides were washed with Phosphate-Buffered Saline (PBS) and then fluorescein isothiocyanate (FITC)-conjugated anti-mouse IgG (ICN Pharmaceuticals,Inc., USA) added to each well and incubated at 37°C for 30 min. Following washes, the slides were examined for virus-specific fluorescence, using an Axioscope fluorescent microscope (Carl Zeiss AG, Germany).

### RNA extraction and RT-PCR

Lung tissues were mechanically homogenized using a FastPrep-24 5G Instrument (MP Biomedicals, USA) with TRI Reagent Solution (Ambion, USA). Total RNA was extracted from lung tissues using a Hybrid R Kit (GeneAll, Korea) according to the manufacturer’s specifications. cDNA was synthesized using M-MLV (Promega, USA) with random hexamers or OSM55 (5'-TAGTAGTAGACTCC-3') [21]. First and nested PCR were performed in 25 μl reaction mixtures containing 200 μM dNTP (Elpis Biotech, USA), 0.25 U of Super-Therm Taq DNA polymerase (JMR Holdings, UK), 1.5 μl of DNA, and 5 pM of each primer. Oligonucleotide primer sequences for the first and nested PCR were specifically designed for HTNV. For the first and nested PCR, the initial denaturation was performed at 94°C for 4 min, followed by six cycles of denaturation at 94°C for 30 sec, annealing at 37°C for 30 sec, elongation at 72°C for 1 min, followed by 32 cycles of denaturation at 94°C for 30 sec, annealing at 42°C for 30 sec, elongation at 72°C for 1 min, and then elongation at 72°C for 5 min. PCR products were extracted using a PCR Purification Kit (Cosmo GENETECH, Korea), and DNA sequencing performed in an Automatic Sequencer, Model ABI 3730XL DNA Analyzer (Applied Biosystems, USA).

### Whole genome sequencing

Viral cDNA was synthesized with random hexamers or OSM55. PCR was performed using specific primer sets covering the whole tripartite genome of HTNV. The PCR program was as follows: a cycle of 95°C for 5 min, 6 cycles of 95°C for 15 sec, 35°C for 30 sec, and 72°C for 20 sec, 30 cycles of 95°C for 15 sec, 42°C for 30 sec, and 72°C for 20 sec and then a cycle of 72°C for 3 min.

### Phylogenetic analysis

The nucleotide sequences of HTNV L, M, and S segments were determined from virus-infected lungs of *A*. *agrarius*. Sequences were aligned and compared with HTNV sequences available in GenBank, using the ClustalW tool in the Lasergene program, version 5 (DNASTAR, USA). For the phylogenetic analysis, the Neighbor-Joining (NJ), Maximum Likelihood (ML), and Bayesian methods (MrBayes 3.2.2 Program) were used. Topologies were evaluated by a bootstrap analysis of 1000 iterations.

### Reassortment analyses

Alignments of HTNV sequences were analyzed using RDP, GENECONV, MAXCHI, CHIMAERA, 3SEQ, BOOTSCAN, and SISCAN in the Recombination Detection Program 4 (RDP4) [22], with concatenated L, M, and S segments. *P*-values under 0.05 were considered statistically significant. All parameters were left at the default RDP settings. The whole genome sequences of HTNV from reassortants, parents, and in- and out-groups were aligned and used to construct maximum likelihood trees of the individual segment in MEGA 5.2 [23].

## Results

### Rodent collection

From 2003–2014, a total of 5,929 striped field mice, *A*. *agrarius*, were collected in Cheorwon, Chuncheon, Hwacheon, Inje, Pyeongchang, Yanggu, and Yangyang in Gangwon province, and Dongducheon, Paju, Pocheon, Phyeongtaek, and Yeoncheon in Gyeonggi province (Table 1). The geographic distribution of rodent trapping sites included military training sites [Dagmar North (DN), Twin Bridge Training Area North (TBTA-N), and Twin Bridge Training Area South (TBTA-S) in Paju; Nightmare Range (NR) in Pocheon; Fire Point 131 (FP131) in Yeoncheon] (Fig 1).

**Table 1:** Total number and screening results of *A*. *agrarius* collected from Gyeonggi and Gangwon provinces in the Republic of Korea, 2003–2014.

Province	District	Year	No. A. agrarius sampled	No. seropositive/ No. sampled (%)	No. RT-PCR positive/ Number seropositive (%)
Gangwon	Cheorwon	2014	67	6/67 (9.0)	5/6 (83.3)
	Hwacheon	2014	63	6/63 (9.5)	3/6 (50)
	Inje	2013	52	2/52 (3.8)	0/2 (0)
		2014	16	1/16 (6.3)	0/1 (0)
	Pyeongchang	2012	53	4/53 (7.5)	0/4 (0)
		2013	87	3/87 (3.4)	0/3 (0)
		2014	34	0/34 (0)	Not determined
	Yanggu	2014	50	10/50 (20)	10/10 (100)
	Yangyang	2014	16	1/16 (6.3)	0/1 (0)
	Chuncheon	2012	26	1/26 (3.8)	0/1 (0)
		2013	64	2/64 (3.1)	0/2 (0)
Gyeonggi	Dongducheon	2012	21	0/21 (0)	Not determined
	Paju	2003	594	100/594 (16.8)	81/100 (81)
		2005	717	200/717 (27.9)	135/200 (67.5)
		2010	628	109/628 (17.4)	92/109 (84.4)
		2012	50	2/50 (4)	2/2 (100)
		2013	22	6/22 (27)	5/6 (83)
		2014	79	20/79 (25.3)	20/20 (100)
	Pocheon	2003	140	16/140 (11.4)	12/16 (75)
		2005	87	12/87 (13.8)	2/12 (16.7)
		2009	1,112	82/1,112 (7.4)	62/82 (75.6)
		2014	29	5/9 (55.6)	4/5 (80)
	Pyeongtaek	2003	24	0/24 (0)	Not determined
		2009	837	17/837 (2)	9/17 (52.9)
		2010	87	1/87 (1.1)	0/1 (0)
		2012	33	0/33 (0)	Not determined
	Yeoncheon	2003	309	58/309 (18.8)	53/58 (91.4)
		2005	593	97/593 (16.4)	38/97 (39.2)
		2013	24	10/24 (42)	8/10 (80)
		2014	15	3/15 (20)	3/3 (100)
Total			5,929	774/5,929 (13.1)	544/756 (70.3)

### Serological and molecular screening for HTNV in wild rodents

**Fig 2:**
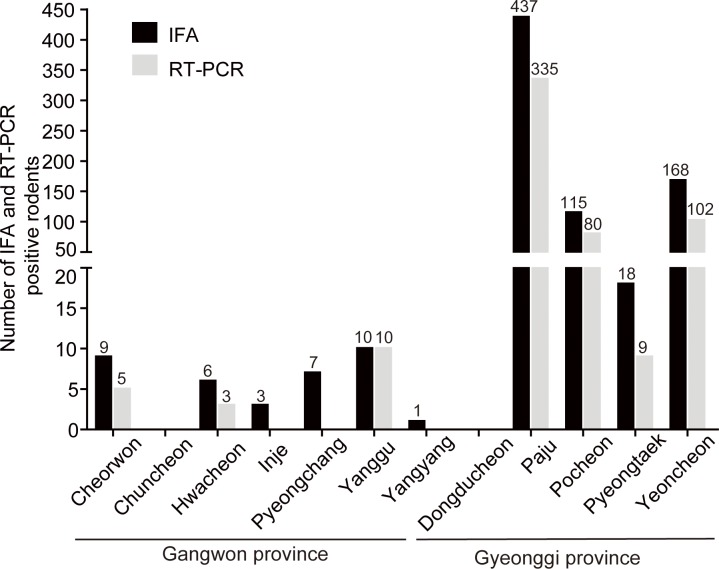
The serological and molecular prevalence of HTNV from *A*. *agrarius* in Gangwon and Gyeonggi provinces. A total of 5,929 *A*. *agrarius* were captured in the Gangwon and Gyeonggi provinces from 2003–2014. A total of 774 *A*. *agrarius* were seropositive for anti-HTNV IgG (shown by black bars). Grey bars represent 544/774 *A*. *agrarius* that were positive for the partial HTNV M segment. Arabic number above the bars indicates the number of IFA and RT-PCR positive rodents, respectively.

To examine the positivity of anti-HTNV IgG, immunofluorescence antibody (IFA) test was performed using sera collected from *A*. *agrarius*. A total of 774/5,929 (13.1%) *A*. *agrarius* were seropositive for anti-HTNV IgG. Among the 774 samples, 36 (4.7%) and 738 (95.3%) of the rodents were captured in Gangwon and Gyeonggi provinces, respectively. Fig 2 showed the number of *A*. *agrarius* which was positive for anti-HTNV IgG from trapping sites. Anti-HTNV IgG in *A*. *agrarius* from Chuncheon and Dongducheon was not detected. Total RNA was extracted from the lung tissues of seropositive samples and determined for the presence of HTNV RNA by RT-PCR. A 393-nucleotide partial sequence of HTNV M segment was recovered from 544/774 (70.3%) of the seropositive samples; including 18 (3.3%) and 526 (96.7%) *A*. *agrarius* in Gangwon and Gyeonggi provinces, respectively (Fig 2).

### Whole genome sequencing

To obtain the whole tripartite genome sequences of HTNV, conventional PCR was performed using multiple primer sets. The whole genome sequencing of the HTNV L, M, and S segments was accomplished for 34 HTNV strains, including 3 strains from Cheorwon, 2 strains from Hwacheon, 6 strains from Yanggu, 6 strains from DN in Paju, 8 strains from TBTA-N and -S in Paju, 3 strains from NR in Pocheon, and 6 strains from FP131 in Yeoncheon (Table 2). The 5´ and 3´ end sequences of HTNV L, M, and S segments were determined by Rapid Amplification of cDNA Ends (RACE) experiments.

**Table 2:** Accession number of the whole genome sequences of HTNV collected from Gyeonggi and Gangwon provinces.

No	Strain	Collection site	Accession number		
			L seg	M seg	S seg
1	Aa03-161	FP131 (Yeoncheon)	KT934956	KT934990	KT935024
2	Aa03-386	FP131 (Yeoncheon)	KT934957	KT934991	KT935025
3	Aa03-387	FP131 (Yeoncheon)	KT934958	KT934992	KT935026
4	Aa05-190	DN (Paju)	KT934959	KT934993	KT935027
5	Aa05-241	DN (Paju)	KT934960	KT934994	KT935028
6	Aa05-249	DN (Paju)	KT934961	KT934995	KT935029
7	Aa05-331	FP131 (Yeoncheon)	KT934962	KT934996	KT935030
8	Aa05-771	FP131 (Yeoncheon)	KT934963	KT934997	KT935031
9	Aa05-775	FP131 (Yeoncheon)	KT934964	KT934998	KT935032
10	Aa09-652	NR (Pocheon)	KT934965	KT934999	KT935033
11	Aa09-948	NR (Pocheon)	KT934966	KT935000	KT935034
12	Aa09-1000	NR (Pocheon)	KT934967	KT935001	KT935035
13	Aa10-123	TBTA-S (Paju)	KT934968	KT935002	KT935036
14	Aa10-288	TBTA-S (Paju)	KT934969	KT935003	KT935037
15	Aa10-434	TBTA-N (Paju)	KT934970	KT935004	KT935038
16	Aa10-518	TBTA-N (Paju)	KT934971	KT935005	KT935039
17	Aa10-561	TBTA-N (Paju)	KT934972	KT935006	KT935040
18	Aa10-679	TBTA-S (Paju)	KT934973	KT935007	KT935041
19	Aa14-172	DN (Paju)	KT934974	KT935008	KT935042
20	Aa14-188	DN (Paju)	KT934975	KT935009	KT935043
21	Aa14-198	DN (Paju)	KT934976	KT935010	KT935044
22	Aa14-204	TBTA-S (Paju)	KT934977	KT935011	KT935045
23	Aa14-207	TBTA-S (Paju)	KT934978	KT935012	KT935046
24	Aa14-266	Hwacheon	KT934979	KT935013	KT935047
25	Aa14-272	Hwacheon	KT934980	KT935014	KT935048
26	Aa14-362	Cheorwon	KT934981	KT935015	KT935049
27	Aa14-368	Cheorwon	KT934982	KT935016	KT935050
28	Aa14-386	Cheorwon	KT934983	KT935017	KT935051
29	Aa14-404	Yanggu	KT934984	KT935018	KT935052
30	Aa14-406	Yanggu	KT934985	KT935019	KT935053
31	Aa14-408	Yanggu	KT934986	KT935020	KT935054
32	Aa14-412	Yanggu	KT934987	KT935021	KT935055
33	Aa14-423	Yanggu	KT934988	KT935022	KT935056
34	Aa14-438	Yanggu	KT934989	KT935023	KT935057

### Phylogenetic analyses

**Fig 3:**
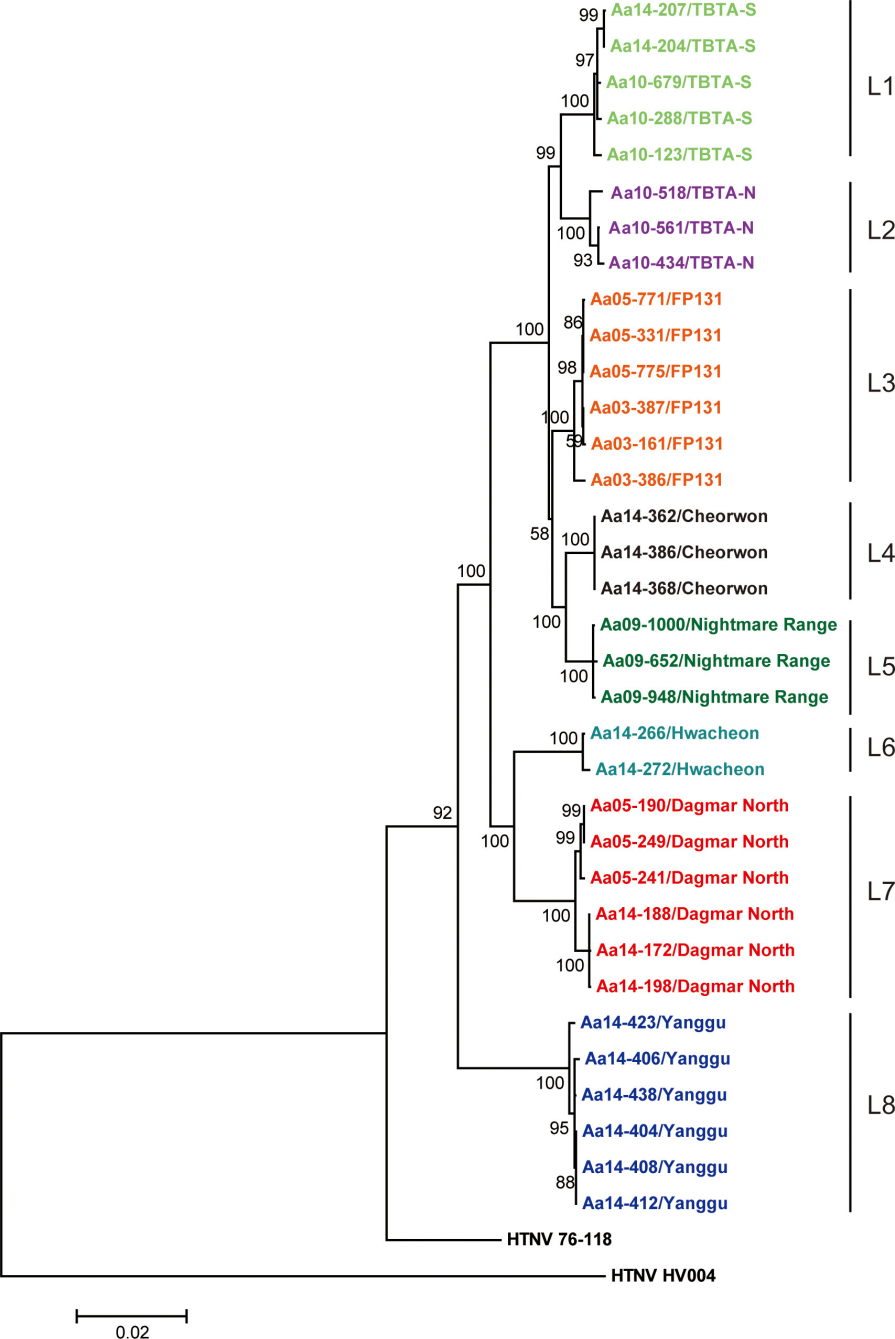
Phylogenetic analysis of whole HTNV L segment genomic sequences in Gangwon and Gyeonggi provinces. The full-length sequences of the HTNV L segment were obtained from lung tissues of *A*. *agrarius* in Gangwon and Gyeonggi provinces. A phylogenetic tree was generated by the ML method. Topologies were evaluated by bootstrap analyses of 1000 iterations. The TN93 (Tamura-Nei)+G model of evolution was used, based on an alignment of the entire nucleotide sequence of the L segment, including strains HTN 76–118 (X55901) and HTN HV004 (JQ083393).

**Fig 4:**
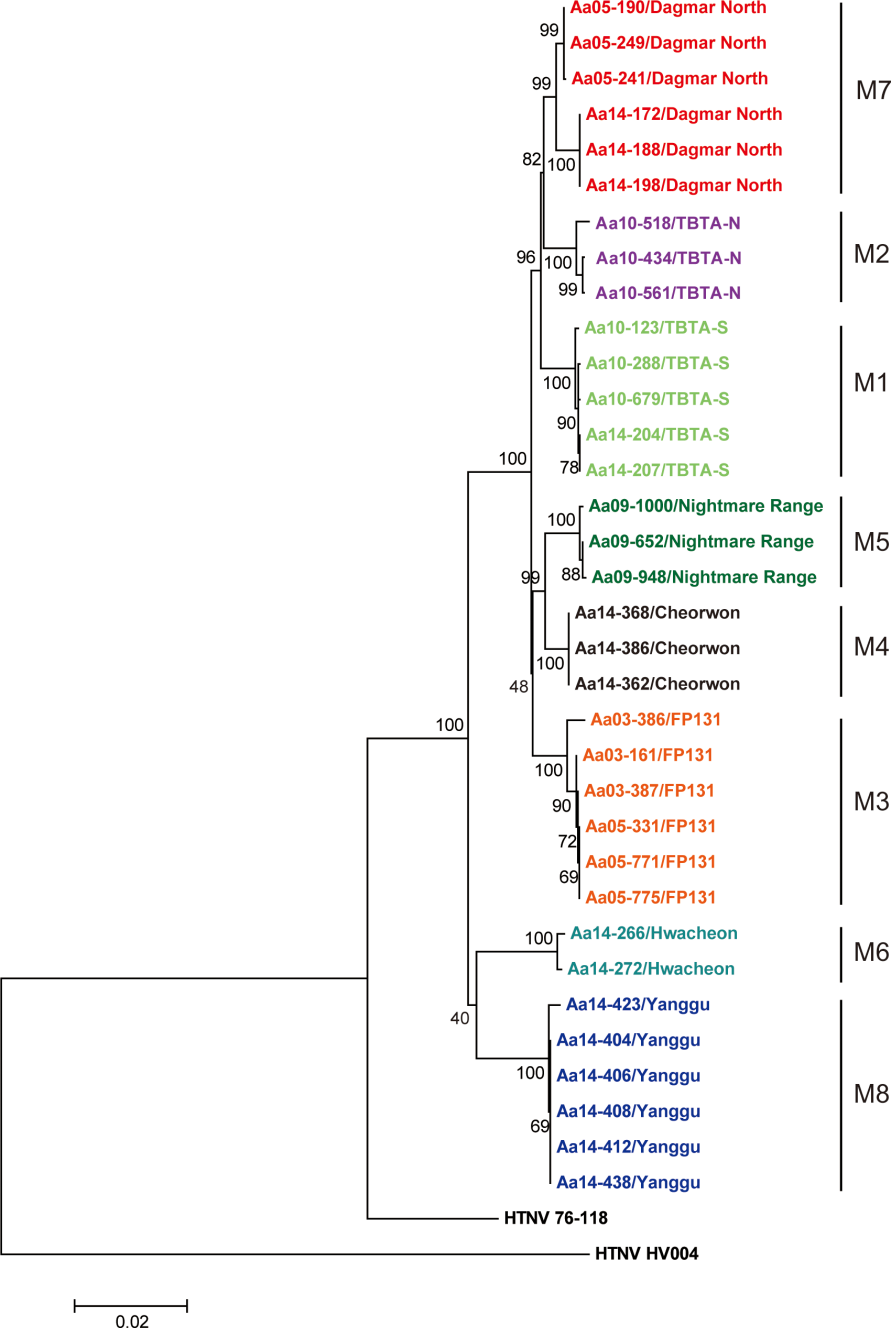
Phylogenetic analysis of whole HTNV M segment genomic sequences in Gangwon and Gyeonggi provinces. The full-length sequences of the HTNV M segment were obtained from lung tissues of *A*. *agrarius* in Gangwon and Gyeonggi provinces. A phylogenetic tree was generated by the ML method. Topologies were evaluated by bootstrap analyses of 1000 iterations. The T92 (Tamura 3-parameter)+G model of evolution was used, based on an alignment of the entire nucleotide sequence of the M segment, including strains HTN 76–118 (M14627) and HTN HV004 (JQ083394).

**Fig 5:**
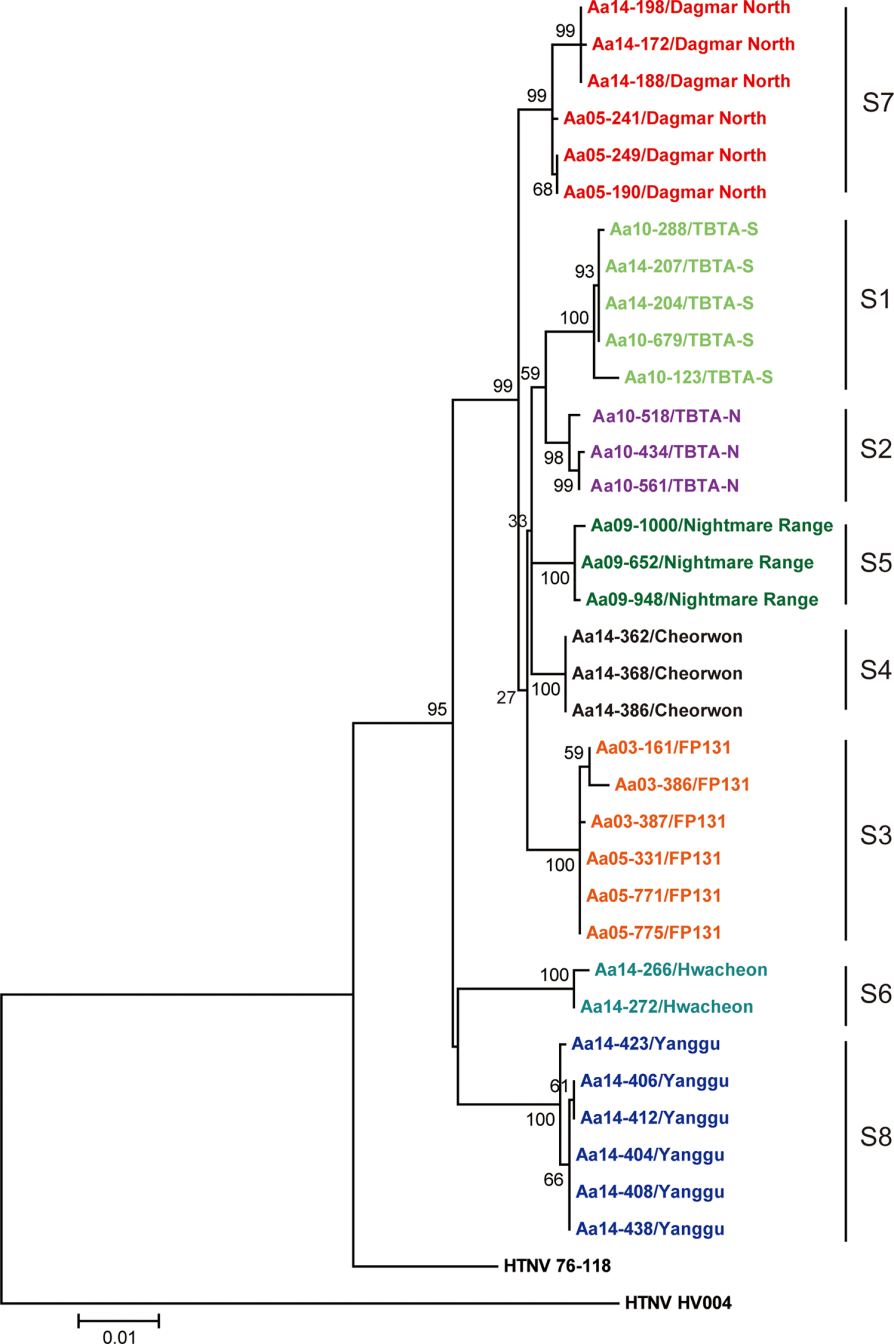
Phylogenetic analysis of whole HTNV S segment genomic sequences in Gangwon and Gyeonggi provinces. The full-length sequences of the HTNV S segment were obtained from lung tissues of *A*. *agrarius* in Gangwon and Gyeonggi provinces. A phylogenetic tree was generated by the ML method. Topologies were evaluated by bootstrap analyses of 1000 iterations. The T92+G model of evolution was used, based on an alignment of the nucleotide sequence of the S segment, including strains HTN 76–118 (M14626) and HTN HV004 (JQ083395).

The full-length sequences of HTNV tripartite RNA genomes were phylogenetically analyzed by Neighbor-Joining (NJ), Maximum Likelihood (ML), and Bayesian methods. The genomic sequences of HTNV L, M, and S segments formed geographic clusters, respectively (Figs 3–5). For the HTNV L segment, the L4 group (Cheorwon) phylogenetically grouped closely with the L5 group (Pocheon). The L8 group (Yanggu) formed a group that was distinct from all other strains from Gyeonggi province. The L6 group (Hwacheon) formed a phylogenetic cluster with the L7 group (DN, Paju). Phylogenetic analyses of the HTNV M and S segments demonstrated that the M4 and S4 groups (Cheorwon) clustered with the M5 and S5 groups (Pocheon). The M and S segments of HTNV strains from Yanggu (M8 and S8) and Hwacheon (M7 and S7) in Gangwon province formed a geographic and distinct clusters from strains obtained in Gyeonggi province. The percent of nucleotide and amino acid sequence homologies of HTNV newly acquired was shown in S1–S3 Tables.

### Reassortment analysis

**Fig 6:**
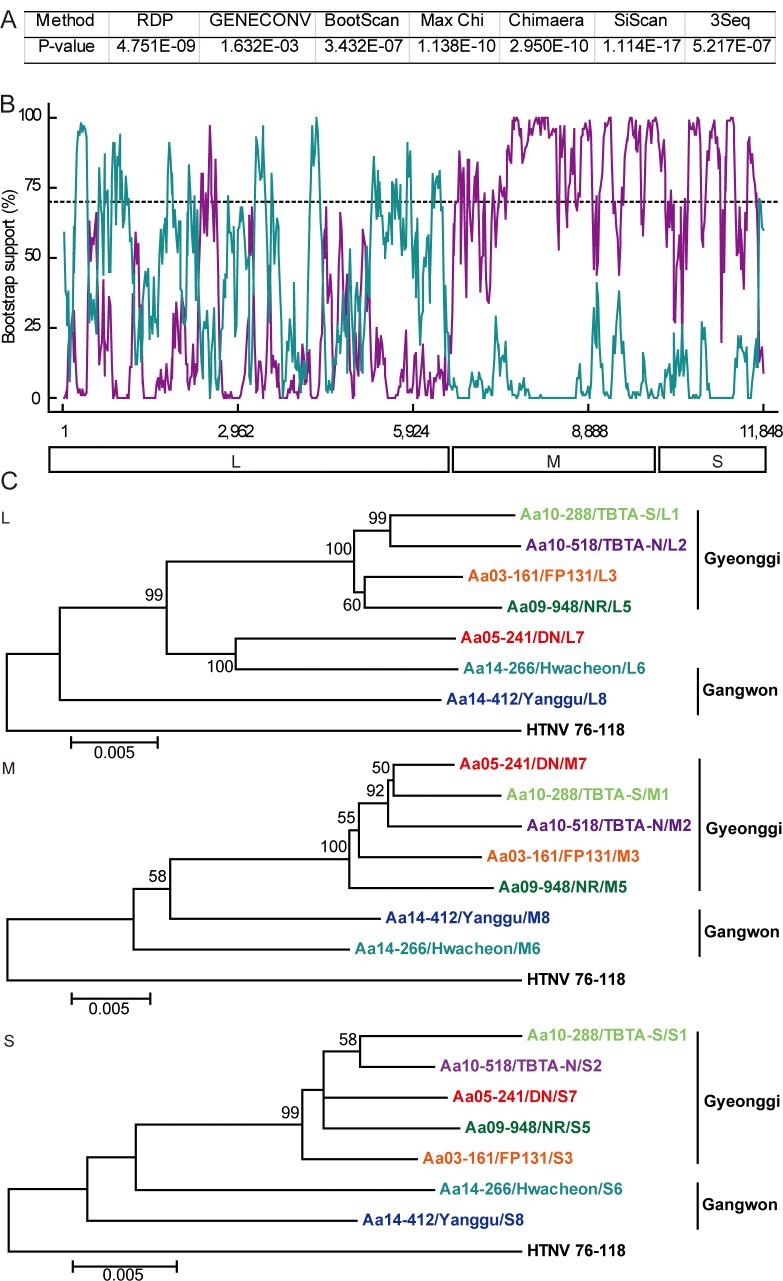
Evidence of a possible genetic reassortment event between HTNV strains in the ROK. (A) *p*-values from the RDP program. (B) The Bootscan plot was based on a pairwise distance model by the RDP4 algorithm. A Bootscan Support Percent of over 70% (cutoff value) was considered significant. Cyan color represents for the comparison of the 7 group (DN, Paju) to the 6 group (Hwacheon); Violet color represents for the comparison of the 7 group (DN, Paju) to the 2 group (TBTA-N, Paju). (C) A reassortant, in-, and out-groups of HTNV were phylogenetically analyzed by the construction of individual ML trees for the L, M, and S segments. The reassortant (the 7 group) is shown in red color. The 6 group (Hwacheon) and the 2 group (TBTA-N, Paju) are indicated by cyan and violet colors, respectively.

Phylogenetic analyses showed that the 7 group (DN, Paju) contained heterogeneous L segment which was clustered with the L6 group (Hwacheon) (Fig 3). However, the M7 and S7 segments of HTNV formed homogeneous clusters with the M2 and S2 groups (TBTA-N, Paju), respectively (Figs 4 and 5). To evaluate the possibility of a reassortment event in the HTNV genomes, the concatenated HTNV tripartite genomes from Gangwon and Gyeonggi provinces were aligned and analyzed using RDP4 software package. Fig 6A shows *P*-values ranging from 1.632E-02 to 1.114E-17 based on the various recombination/reassortment analysis programs. The RDP recombination consensus score (RDPRCS) of the 7 group (DN, Paju) is 0.522. Fig 6B shows high percent of bootstrap support for a grouping of the HTNV M and S segments from the 7 group (DN, Paju) and the 2 group (TBTA-N, Paju), whereas the L segment shows a high similarity between the 7 group (DN, Paju) and the 6 group (Hwacheon). Eight HTNV stains representing reassortants, parents, and in- and out- groups were used to generate maximum likelihood trees of the HTNV L, M, and S segments, respectively (Fig 6C). These results suggest that the 7 group (DN, Paju) were reassortants in nature based on the heterogeneity of the L segment, as the L segment formed a lineage with the 6 group (Hwacheon) in Gangwon province, while the M and S segments clustered with the 2 group (TBTA-N, Paju) in Gyeonggi province.

## Discussion

HFRS is highly endemic in Gangwon and Gyeonggi provinces, ROK, affecting both military personnel and civilians. For decades, we conducted epidemiological and phylogeographic analyses of HTNV in Gyeonggi province. Recent clinical cases of HFRS in Gangwon province, including one deceased patient in 2013, prompted us to investigate the geographic distribution and diversity of HTNV in Gangwon province [24].

A total of 5,929 *A*. *agrarius* were collected in the endemic areas of Gangwon and Gyeonggi provinces from 2003–2014. IFA tests showed that 774 (13.1%) *A*. *agrarius* were seropositive for anti-HTNV IgG. HTNV-specific RT-PCR showed that 544/774 (70.3%) *A*. *agrarius* were positive for a partial HTNV M segment. Based on the serological and molecular tests, there was a lower prevalence of HTNV infections among *A*. *agrarius* in Gangwon province than in Gyeonggi province. Although the number of *A*. *agrarius* was much lower for Gangwon province, these data suggest that Gyeonggi province poses significantly higher HFRS health risks than Gangwon province [8, 9, 14, 15]. To support this, the incidence of HFRS patients in Gangwon province was lower than that in Gyeonggi province, demonstrating a correlation between rodent seroprevalence and human disease data [25].

Using the complete genomic sequences of 34 HTNV strains newly obtained from eight different trapping sites, the phylogenetic trees of HTNV L, M, and S segments demonstrated well-supported geographic clusters and showed the genetic diversity in the restricted areas. Diversification of HTNV, the Old World hantavirus, has been observed in China, a high HFRS-endemic area [20]. In Guizhou, high level of the molecular diversity of HTNV strains were observed. The ADNV, a New World hantavirus from southern South America, also exhibited high molecular diversity of the M segment, resulting in five different lineages based on their geographic origins in Argentina and Chile [26, 27]. Consistent with these observations, HTNV strains in the restricted areas of the ROK showed a high molecular diversity representing geographic distinct clusters.

Reassortment, recombination, and genetic drift are molecular genetic mechanisms that confer the genetic diversity in RNA viruses in nature [28]. Segmented RNA viruses preferentially give rise to genetic reassortment rather than recombination. A reassortment event predicted by RDP4 was considered significant if it satisfied at least 2 criteria with a *P-value (p) <*0.05 and the RDPRCS was >0.6 [29]. When *P* <0.05 and the RDPRCS was between 0.4 and 0.6, the genetic event was considered possible. An RDPRCS under 0.4 with *P* <0.05 was a cause to reject the genetic event. The HTNV strains (the 7 group) from DN were likely reassortants since these strains showed *P* <0.05 and an RDPRCS of 0.522 (HTNV from Hwacheon, 0.322; HTNV from TBTA-N, 0.156).

The genetic reassortment of bunyaviruses in nature has been previously reported [30, 31]. Reassortments of SNV have more commonly been described in M segments than S or L segments in nature and *in vitro* [18, 31]. The L, M, and S segments of hantaviruses encode the viral RNA-dependent RNA-polymerase (the L protein), two surface glycoproteins (Gn and Gc), and the nucleocapsid (N), respectively [32]. The less common reassortants of L or S segments than M segment might be associated with the function of these viral proteins. The maintenance of L and S segments might be beneficial for replication, transcription, and assembly, resulting in the production of the progeny possessed appropriate viral fitness. Still, given our limited understanding of this genetic mechanism, the existence of reassortants that contain different combinations of segments should not be ignored. Infection with Guaroa virus generated the L segment reassortant, whereas the S segment reassortant was observed between Bunyamwera and California encephalitis viruses [33]. In this study, the reassortment analysis demonstrated that HTNV (the 7 group) from DN in Paju showed heterogeneous L segment, but homogeneous M and S segments. Whether the exchange of L segment is a determinant of the fitness and pathogenesis of HTNV remains to be studied.

In conclusion, we report the phylogeographic analysis of full-length HTNV sequences from *A*. *agrarius* collected in HFRS-endemic areas of Gangwon and Gyeonggi provinces, ROK. These results demonstrate the geographic diversity and a possible reassortment of HTNV in nature. This study greatly increases our understanding of the genetic diversity and molecular evolution of HTNV in the hantavirus-endemic areas. The whole sequences of HTNV tripartite genomes will provide a database for the phylogeographic analysis and surveillance of hantavirus infection from HFRS patients and natural reservoir hosts.

## Supporting Information

S1 Table Percent of nucleotide and amino acid sequence homology among the L segment of Hantaan Virus (HTNV). (DOCX)

S2 Table Percent of nucleotide and amino acid sequence homology among the M segment of Hantaan Virus (HTNV). (DOCX)

S3 Table Percent of nucleotide and amino acid sequence homology among the S segment of Hantaan Virus (HTNV). (DOCX)
