# Supplementary material for: Genetic Diversity and Reassortment of Hantaan Virus Tripartite RNA Genomes in Nature, the Republic of Korea
Source: PLoS Negl Trop Dis. 2016 Jun 17;10(6):e0004650. doi: 10.1371/journal.pntd.0004650 (PMC4912082; doi:10.1371/journal.pntd.0004650)
Supplement: S2 Table — (DOCX) [file pntd.0004650.s002.docx]

S2 Table. Percent of nucleotide and amino acid sequence homology among the M segment of Hantaan Virus (HTNV).

|  | 1 | 2 | 3 | 4 | 5 | 6 | 7 | 8 | 9 | 10 | 11 | 12 | 13 | 14 | 15 | 16 | 17 | 18 | 19 | 20 | 21 | 22 | 23 | 24 | 25 | 26 | 27 | 28 | 29 | 30 | 31 | 32 | 33 | 34 | 35 | 36 |
| --- | --- | --- | --- | --- | --- | --- | --- | --- | --- | --- | --- | --- | --- | --- | --- | --- | --- | --- | --- | --- | --- | --- | --- | --- | --- | --- | --- | --- | --- | --- | --- | --- | --- | --- | --- | --- |
| Nucleotide identities (%) | | | | | | | | | | | | | | | | | | | | | | | | | | | | | | | | | | | | |
| 1 | *** | 99.5 | 100 | 98.7 | 98.6 | 98.7 | 100 | 99.9 | 100 | 98.4 | 98.3 | 98.4 | 98.4 | 98.4 | 98.3 | 98.3 | 98.3 | 98.4 | 98.4 | 98.4 | 98.4 | 98.4 | 98.4 | 96.7 | 96.8 | 98.6 | 98.6 | 98.6 | 96.8 | 96.8 | 96.8 | 96.8 | 96.7 | 96.8 | 94.5 | 83.9 |
| 2 | 99.7 | *** | 99.5 | 98.5 | 98.5 | 98.5 | 99.5 | 99.5 | 99.5 | 98.2 | 98.2 | 98.2 | 98.3 | 98.2 | 98.2 | 98.1 | 98.2 | 98.2 | 98.2 | 98.2 | 98.2 | 98.2 | 98.2 | 96.7 | 96.7 | 98.5 | 98.5 | 98.5 | 96.7 | 96.7 | 96.7 | 96.7 | 96.6 | 96.7 | 94.4 | 83.9 |
| 3 | 100 | 99.7 | *** | 98.7 | 98.6 | 98.7 | 100 | 99.9 | 100 | 98.4 | 98.3 | 98.4 | 98.4 | 98.4 | 98.3 | 98.3 | 98.3 | 98.4 | 98.4 | 98.4 | 98.4 | 98.4 | 98.4 | 96.7 | 96.8 | 98.6 | 98.6 | 98.6 | 96.8 | 96.8 | 96.8 | 96.8 | 96.7 | 96.8 | 94.5 | 83.9 |
| 4 | 99.7 | 99.6 | 99.7 | *** | 100 | 100 | 98.6 | 98.6 | 98.6 | 98.5 | 98.5 | 98.5 | 99 | 99 | 98.9 | 98.8 | 98.9 | 99 | 99.4 | 99.4 | 99.4 | 99 | 99 | 96.8 | 96.8 | 98.8 | 98.8 | 98.8 | 97 | 97 | 97 | 97 | 96.8 | 97 | 94.5 | 83.8 |
| 5 | 99.7 | 99.6 | 99.7 | 100 | *** | 100 | 98.6 | 98.6 | 98.6 | 98.5 | 98.5 | 98.5 | 99 | 98.9 | 98.9 | 98.8 | 98.9 | 98.9 | 99.4 | 99.4 | 99.4 | 98.9 | 98.9 | 96.8 | 96.8 | 98.8 | 98.8 | 98.8 | 97 | 97 | 97 | 97 | 96.8 | 97 | 94.5 | 83.8 |
| 6 | 99.7 | 99.6 | 99.7 | 100 | 100 | *** | 98.6 | 98.6 | 98.6 | 98.5 | 98.5 | 98.5 | 99 | 99 | 98.9 | 98.8 | 98.9 | 99 | 99.4 | 99.4 | 99.4 | 99 | 99 | 96.8 | 96.8 | 98.8 | 98.8 | 98.8 | 97 | 97 | 97 | 97 | 96.8 | 97 | 94.5 | 83.8 |
| 7 | 100 | 99.7 | 100 | 99.7 | 99.7 | 99.7 | *** | 100 | 100 | 98.3 | 98.3 | 98.3 | 98.4 | 98.3 | 98.3 | 98.2 | 98.3 | 98.3 | 98.3 | 98.3 | 98.3 | 98.3 | 98.3 | 96.7 | 96.8 | 98.6 | 98.6 | 98.6 | 96.8 | 96.8 | 96.8 | 96.8 | 96.6 | 96.8 | 94.5 | 83.9 |
| 8 | 100 | 99.7 | 100 | 99.7 | 99.7 | 99.7 | 100 | *** | 100 | 98.3 | 98.3 | 98.3 | 98.4 | 98.3 | 98.3 | 98.2 | 98.3 | 98.3 | 98.3 | 98.3 | 98.3 | 98.3 | 98.3 | 96.7 | 96.7 | 98.6 | 98.6 | 98.6 | 96.8 | 96.8 | 96.8 | 96.8 | 96.6 | 96.8 | 94.4 | 83.9 |
| 9 | 100 | 99.7 | 100 | 99.7 | 99.7 | 99.7 | 100 | 100 | *** | 98.3 | 98.3 | 98.3 | 98.4 | 98.3 | 98.3 | 98.2 | 98.3 | 98.3 | 98.3 | 98.3 | 98.3 | 98.3 | 98.3 | 96.7 | 96.8 | 98.6 | 98.6 | 98.6 | 96.8 | 96.8 | 96.8 | 96.8 | 96.6 | 96.8 | 94.5 | 83.9 |
| 10 | 99.9 | 99.8 | 99.9 | 99.8 | 99.8 | 99.8 | 99.9 | 99.9 | 99.9 | *** | 99.9 | 99.9 | 98.3 | 98.2 | 98.2 | 98.2 | 98.2 | 98.2 | 98.2 | 98.2 | 98.2 | 98.2 | 98.2 | 96.6 | 96.6 | 98.9 | 98.9 | 98.9 | 96.9 | 96.9 | 96.9 | 96.9 | 96.7 | 96.9 | 94.2 | 83.8 |
| 11 | 99.8 | 99.7 | 99.8 | 99.7 | 99.7 | 99.7 | 99.8 | 99.8 | 99.8 | 99.9 | *** | 99.8 | 98.2 | 98.2 | 98.1 | 98.1 | 98.1 | 98.2 | 98.2 | 98.2 | 98.2 | 98.2 | 98.2 | 96.5 | 96.5 | 98.9 | 98.9 | 98.9 | 96.8 | 96.8 | 96.8 | 96.8 | 96.7 | 96.8 | 94.2 | 83.8 |
| 12 | 99.9 | 99.8 | 99.9 | 99.8 | 99.8 | 99.8 | 99.9 | 99.9 | 99.9 | 100 | 99.9 | *** | 98.3 | 98.2 | 98.2 | 98.2 | 98.2 | 98.2 | 98.2 | 98.2 | 98.2 | 98.2 | 98.2 | 96.6 | 96.6 | 98.9 | 98.9 | 98.9 | 96.9 | 96.9 | 96.9 | 96.9 | 96.7 | 96.9 | 94.2 | 83.8 |
| 13 | 99.9 | 99.8 | 99.9 | 99.8 | 99.8 | 99.8 | 99.9 | 99.9 | 99.9 | 100 | 99.9 | 100 | *** | 99.9 | 98.6 | 98.5 | 98.6 | 99.9 | 98.7 | 98.7 | 98.7 | 99.9 | 99.9 | 96.7 | 96.7 | 98.5 | 98.5 | 98.5 | 96.9 | 96.9 | 96.9 | 96.9 | 96.7 | 96.9 | 94.6 | 83.9 |
| 14 | 99.9 | 99.8 | 99.9 | 99.8 | 99.8 | 99.8 | 99.9 | 99.9 | 99.9 | 100 | 99.9 | 100 | 100 | *** | 98.5 | 98.5 | 98.6 | 99.9 | 98.6 | 98.6 | 98.6 | 99.9 | 99.9 | 96.7 | 96.7 | 98.5 | 98.5 | 98.5 | 96.9 | 96.9 | 96.9 | 96.9 | 96.7 | 96.9 | 94.6 | 84 |
| 15 | 99.6 | 99.5 | 99.6 | 99.5 | 99.5 | 99.5 | 99.6 | 99.6 | 99.6 | 99.6 | 99.6 | 99.6 | 99.6 | 99.6 | *** | 99.6 | 99.9 | 98.5 | 98.7 | 98.7 | 98.7 | 98.5 | 98.5 | 96.5 | 96.5 | 98.4 | 98.4 | 98.4 | 96.7 | 96.7 | 96.7 | 96.7 | 96.5 | 96.7 | 94.3 | 83.7 |
| 16 | 99.7 | 99.6 | 99.7 | 99.6 | 99.6 | 99.6 | 99.7 | 99.7 | 99.7 | 99.8 | 99.7 | 99.8 | 99.8 | 99.8 | 99.5 | *** | 99.6 | 98.5 | 98.6 | 98.6 | 98.6 | 98.5 | 98.5 | 96.5 | 96.5 | 98.3 | 98.3 | 98.3 | 96.7 | 96.7 | 96.7 | 96.7 | 96.5 | 96.7 | 94.4 | 83.8 |
| 17 | 99.6 | 99.6 | 99.6 | 99.6 | 99.6 | 99.6 | 99.6 | 99.6 | 99.6 | 99.7 | 99.6 | 99.7 | 99.7 | 99.7 | 99.9 | 99.6 | *** | 98.5 | 98.7 | 98.7 | 98.7 | 98.5 | 98.5 | 96.6 | 96.6 | 98.4 | 98.4 | 98.4 | 96.8 | 96.8 | 96.8 | 96.8 | 96.6 | 96.8 | 94.3 | 83.8 |
| 18 | 99.9 | 99.8 | 99.9 | 99.8 | 99.8 | 99.8 | 99.9 | 99.9 | 99.9 | 100 | 99.9 | 100 | 100 | 100 | 99.6 | 99.8 | 99.7 | *** | 98.6 | 98.6 | 98.6 | 99.9 | 99.9 | 96.7 | 96.7 | 98.5 | 98.5 | 98.5 | 96.9 | 96.9 | 96.9 | 96.9 | 96.7 | 96.9 | 94.6 | 84 |
| 19 | 99.6 | 99.5 | 99.6 | 99.8 | 99.8 | 99.8 | 99.6 | 99.6 | 99.6 | 99.6 | 99.6 | 99.6 | 99.6 | 99.6 | 99.3 | 99.5 | 99.4 | 99.6 | *** | 100 | 100 | 98.6 | 98.6 | 96.5 | 96.5 | 98.6 | 98.6 | 98.6 | 96.7 | 96.7 | 96.7 | 96.7 | 96.5 | 96.7 | 94.1 | 83.7 |
| 20 | 99.6 | 99.5 | 99.6 | 99.8 | 99.8 | 99.8 | 99.6 | 99.6 | 99.6 | 99.6 | 99.6 | 99.6 | 99.6 | 99.6 | 99.3 | 99.5 | 99.4 | 99.6 | 100 | *** | 100 | 98.6 | 98.6 | 96.5 | 96.5 | 98.6 | 98.6 | 98.6 | 96.7 | 96.7 | 96.7 | 96.7 | 96.5 | 96.7 | 94.1 | 83.7 |
| 21 | 99.6 | 99.5 | 99.6 | 99.8 | 99.8 | 99.8 | 99.6 | 99.6 | 99.6 | 99.6 | 99.6 | 99.6 | 99.6 | 99.6 | 99.3 | 99.5 | 99.4 | 99.6 | 100 | 100 | *** | 98.6 | 98.6 | 96.5 | 96.5 | 98.6 | 98.6 | 98.6 | 96.7 | 96.7 | 96.7 | 96.7 | 96.5 | 96.7 | 94.1 | 83.7 |
| 22 | 99.9 | 99.8 | 99.9 | 99.8 | 99.8 | 99.8 | 99.9 | 99.9 | 99.9 | 100 | 99.9 | 100 | 100 | 100 | 99.6 | 99.8 | 99.7 | 100 | 99.6 | 99.6 | 99.6 | *** | 100 | 96.7 | 96.7 | 98.5 | 98.5 | 98.5 | 96.8 | 96.8 | 96.8 | 96.8 | 96.7 | 96.8 | 94.6 | 84 |
| 23 | 99.9 | 99.8 | 99.9 | 99.8 | 99.8 | 99.8 | 99.9 | 99.9 | 99.9 | 100 | 99.9 | 100 | 100 | 100 | 99.6 | 99.8 | 99.7 | 100 | 99.6 | 99.6 | 99.6 | 100 | *** | 96.7 | 96.7 | 98.5 | 98.5 | 98.5 | 96.8 | 96.8 | 96.8 | 96.8 | 96.7 | 96.8 | 94.6 | 84 |
| 24 | 99.2 | 99.1 | 99.2 | 99.1 | 99.1 | 99.1 | 99.2 | 99.2 | 99.2 | 99.3 | 99.2 | 99.3 | 99.3 | 99.3 | 98.9 | 99.1 | 99 | 99.3 | 98.9 | 98.9 | 98.9 | 99.3 | 99.3 | *** | 99.8 | 96.7 | 96.7 | 96.7 | 97.2 | 97.2 | 97.2 | 97.2 | 97 | 97.2 | 94.9 | 83.9 |
| 25 | 99.3 | 99.2 | 99.3 | 99.2 | 99.2 | 99.2 | 99.3 | 99.3 | 99.3 | 99.4 | 99.3 | 99.4 | 99.4 | 99.4 | 99 | 99.2 | 99.1 | 99.4 | 99 | 99 | 99 | 99.4 | 99.4 | 99.9 | *** | 96.7 | 96.7 | 96.7 | 97.2 | 97.2 | 97.2 | 97.2 | 97.1 | 97.2 | 94.9 | 84 |
| 26 | 99.7 | 99.6 | 99.7 | 99.6 | 99.6 | 99.6 | 99.7 | 99.7 | 99.7 | 99.8 | 99.7 | 99.8 | 99.8 | 99.8 | 99.5 | 99.6 | 99.6 | 99.8 | 99.5 | 99.5 | 99.5 | 99.8 | 99.8 | 99.1 | 99.2 | *** | 100 | 100 | 97 | 97 | 97 | 97 | 96.9 | 97 | 94.6 | 83.8 |
| 27 | 99.7 | 99.6 | 99.7 | 99.6 | 99.6 | 99.6 | 99.7 | 99.7 | 99.7 | 99.8 | 99.7 | 99.8 | 99.8 | 99.8 | 99.5 | 99.6 | 99.6 | 99.8 | 99.5 | 99.5 | 99.5 | 99.8 | 99.8 | 99.1 | 99.2 | 100 | *** | 100 | 97 | 97 | 97 | 97 | 96.9 | 97 | 94.6 | 83.8 |
| 28 | 99.7 | 99.6 | 99.7 | 99.6 | 99.6 | 99.6 | 99.7 | 99.7 | 99.7 | 99.8 | 99.7 | 99.8 | 99.8 | 99.8 | 99.5 | 99.6 | 99.6 | 99.8 | 99.5 | 99.5 | 99.5 | 99.8 | 99.8 | 99.1 | 99.2 | 100 | 100 | *** | 97 | 97 | 97 | 97 | 96.9 | 97 | 94.6 | 83.8 |
| 29 | 99.2 | 99.1 | 99.2 | 99.1 | 99.1 | 99.1 | 99.2 | 99.2 | 99.2 | 99.3 | 99.2 | 99.3 | 99.3 | 99.3 | 98.9 | 99.1 | 99 | 99.3 | 98.9 | 98.9 | 98.9 | 99.3 | 99.3 | 99.6 | 99.7 | 99.1 | 99.1 | 99.1 | *** | 100 | 100 | 100 | 99.8 | 100 | 94.9 | 84.2 |
| 30 | 99.2 | 99.1 | 99.2 | 99.1 | 99.1 | 99.1 | 99.2 | 99.2 | 99.2 | 99.3 | 99.2 | 99.3 | 99.3 | 99.3 | 98.9 | 99.1 | 99 | 99.3 | 98.9 | 98.9 | 98.9 | 99.3 | 99.3 | 99.6 | 99.7 | 99.1 | 99.1 | 99.1 | 100 | *** | 100 | 100 | 99.8 | 100 | 94.9 | 84.2 |
| 31 | 99.2 | 99.1 | 99.2 | 99.1 | 99.1 | 99.1 | 99.2 | 99.2 | 99.2 | 99.3 | 99.2 | 99.3 | 99.3 | 99.3 | 98.9 | 99.1 | 99 | 99.3 | 98.9 | 98.9 | 98.9 | 99.3 | 99.3 | 99.6 | 99.7 | 99.1 | 99.1 | 99.1 | 100 | 100 | *** | 100 | 99.8 | 100 | 94.9 | 84.2 |
| 32 | 99.2 | 99.1 | 99.2 | 99.1 | 99.1 | 99.1 | 99.2 | 99.2 | 99.2 | 99.3 | 99.2 | 99.3 | 99.3 | 99.3 | 98.9 | 99.1 | 99 | 99.3 | 98.9 | 98.9 | 98.9 | 99.3 | 99.3 | 99.6 | 99.7 | 99.1 | 99.1 | 99.1 | 100 | 100 | 100 | *** | 99.8 | 100 | 94.9 | 84.2 |
| 33 | 99.1 | 99 | 99.1 | 99 | 99 | 99 | 99.1 | 99.1 | 99.1 | 99.2 | 99.1 | 99.2 | 99.2 | 99.2 | 98.9 | 99 | 98.9 | 99.2 | 98.9 | 98.9 | 98.9 | 99.2 | 99.2 | 99.6 | 99.6 | 99 | 99 | 99 | 99.9 | 99.9 | 99.9 | 99.9 | *** | 99.8 | 94.8 | 84.1 |
| 34 | 99.2 | 99.1 | 99.2 | 99.1 | 99.1 | 99.1 | 99.2 | 99.2 | 99.2 | 99.3 | 99.2 | 99.3 | 99.3 | 99.3 | 98.9 | 99.1 | 99 | 99.3 | 98.9 | 98.9 | 98.9 | 99.3 | 99.3 | 99.6 | 99.7 | 99.1 | 99.1 | 99.1 | 100 | 100 | 100 | 100 | 99.9 | *** | 94.9 | 84.2 |
| 35 | 98.6 | 98.5 | 98.6 | 98.5 | 98.5 | 98.5 | 98.6 | 98.6 | 98.6 | 98.7 | 98.6 | 98.7 | 98.7 | 98.7 | 98.5 | 98.5 | 98.4 | 98.7 | 98.4 | 98.4 | 98.4 | 98.7 | 98.7 | 99 | 99.1 | 98.5 | 98.5 | 98.5 | 99 | 99 | 99 | 99 | 98.9 | 99 | *** | 84 |
| 36 | 97.2 | 97.1 | 97.2 | 97.1 | 97.1 | 97.1 | 97.2 | 97.2 | 97.2 | 97.3 | 97.2 | 97.3 | 97.3 | 97.3 | 96.9 | 97.1 | 97 | 97.3 | 97 | 97 | 97 | 97.3 | 97.3 | 97.3 | 97.4 | 97.1 | 97.1 | 97.1 | 97.3 | 97.3 | 97.3 | 97.3 | 97.2 | 97.3 | 97.1 | *** |
| Amino acid identities (%) | | | | | | | | | | | | | | | | | | | | | | | | | | | | | | | | | | | | |

1. Aa03-161, 2. Aa03-386, 3. Aa03-387, 4. Aa05-190, 5. Aa05-241, 6. Aa05-249, 7. Aa05-331, 8. Aa05-771, 9. Aa05-775, 10. Aa09-652, 11. Aa09-948, 12. Aa09-1000,

13. Aa10-123, 14. Aa10-288, 15. Aa10-434, 16. Aa10-518, 17. Aa10-561, 18. Aa10-679, 19. Aa14-172, 20. Aa14-188, 21. Aa14-198, 22. Aa14-204, 23. Aa14-207,

24. Aa14-266, 25. Aa14-272, 26. Aa14-362, 27. Aa14-368, 28. Aa14-386, 29. Aa14-404, 30. Aa14-406, 31. Aa14-408, 32. Aa14-412, 33. Aa14-423, 34. Aa14-438,

35. HTNV76-118, 36. HV004.
